# Do Biliary Complications after Proton Beam Therapy for Perihilar Hepatocellular Carcinoma Matter?

**DOI:** 10.3390/cancers12092395

**Published:** 2020-08-24

**Authors:** Gyu Sang Yoo, Jeong Il Yu, Hee Chul Park, Dongho Hyun, Woo Kyoung Jeong, Ho Yeong Lim, Moon Seok Choi, Sang Yun Ha

**Affiliations:** 1 Department of Radiation Oncology, Samsung Medical Center, Sungkyunkwan University School of Medicine, Seoul 06351, Korea; demian81@skku.edu (G.S.Y.); royuji651@skku.edu (J.I.Y.); 2 Department of Radiology, Samsung Medical Center, Sungkyunkwan University School of Medicine, Seoul 06351, Korea; mesentery.hyun@samsung.com (D.H.); jeongwk@skku.edu (W.K.J.); 3 Department of Internal Medicine (Division of Hematology-Oncology), Samsung Medical Center, Sungkyunkwan University School of Medicine, Seoul 06351, Korea; hoylim@skku.edu; 4 Department of Internal Medicine (Division of Gastroenterology), Samsung Medical Center, Sungkyunkwan University School of Medicine, Seoul 06351, Korea; mschoi@skku.edu; 5 Department of Pathology, Samsung Medical Center, Sungkyunkwan University School of Medicine, Seoul 06351, Korea; sangyun.ha@skku.edu

**Keywords:** proton beam therapy, hepatocellular carcinoma, perihilar region, biliary complication, local control, overall survival

## Abstract

We aimed to evaluate the biliary complications and efficacy of proton beam therapy (PBT) for hepatocellular carcinoma (HCC). We retrospectively analyzed 167 patients who received PBT with ≥ 75 GyRBE of biological effective dose with 𝛼/β = 10 for primary HCC. The perihilar region was defined as a 1-cm area extending from the right, left, and common hepatic ducts, including the gallbladder and cystic duct. PBT-related biliary complications were defined as follows: significant elevation in bilirubin level to > 3.0 mg/dL; elevation to more than twice of the baseline level after the completion of PBT; or newly developed radiological biliary abnormalities, which were not caused by HCC progression, comorbidities, or other treatments. Eighty (47.9%) had perihilar HCC. PBT-related events occurred in seven (4.2%), three of whom had perihilar HCC. Radiologic biliary abnormalities developed in 12 patients (7.2%); however, no events were PBT-related. All patients who experienced PBT-related biliary complications had underlying liver cirrhosis. The albumin-bilirubin grade was identified as an independent factor associated with PBT-related biliary complications. PBT at the current dose showed a low rate of PBT-related biliary complications even for patients with perihilar HCC. PBT for HCC patients with risk factors requires attention to reduce PBT-related biliary complications.

## 1. Introduction

Hepatocellular carcinoma (HCC) is the most common hepatic malignancy and the fifth most common cancer worldwide [1]. The Barcelona Clinic Liver Cancer (BCLC) system recommends various treatments for HCC, including surgical resection, liver transplantation, radiofrequency ablation (RFA), trans-arterial chemoembolization (TACE), and other systemic treatments [2]. However, there remains a need for other effective local modalities for advanced disease or early cases with a unsuitable tumor location or poor liver condition for curative resection or RFA [3]. Radiation therapy (RT) is non-invasive, local modality with fewer limitations regarding patients’ medical status and tumor characteristics, including the tumor size or location. Therefore, the use of RT has been increased as a combined or alternative modality to standard therapy [4,5,6]. Furthermore, with advances in RT technologies, including stereotactic body RT and proton beam therapy (PBT), the highly accurate delivery of high-dose radiation to primary HCC has resulted in excellent local control after RT without an increased risk of radiation-induced liver disease, which had previously been the main concern of liver-directed RT [7,8,9,10,11]. Among the complications of treatments for primary HCC, biliary complications including bile duct stricture or biloma are important issues. Biliary complications can be vulnerable or even fatal to HCC patients with underlying liver disease because of the difficulty in their managements. Some studies have suggested that biliary complications could be related to RT. This concern is greater in RT for primary HCC in perihilar locations because biliary damage due to radiation may induce liver failure or biliary sepsis. However, data regarding radiation-induced biliary complication are limited [12,13,14,15]. These data are even more limited for PBT, which has higher relative biological effectiveness, potentially leading to frequent and severe biliary complications. Our institute has performed high-dose PBT in a relatively high number of patients with primary HCC in a short period. Therefore, based on our experience, we aimed to evaluate the risk of and relevant risk factors for biliary complications after high-dose PBT for primary HCC.

## 2. Results

The median patients’ age was 62 years (range, 35–91 years). The median follow-up duration was 14 months (range, 1–29 months). The characteristics of patients according to tumor location are summarized in Table 1.

The proportions of patients with albumin-bilirubin (ALBI) grade 1, better performance status, HCC with a maximum diameter of 2 cm or less, earlier BCLC stage, and higher prescribed dose for PBT were significantly higher in the non-perihilar HCC group. However, there were no significant differences in the PBT beam module or respiratory control method between the groups. The mean clinical target volume (CTV) and mean liver volume were significantly larger in perihilar group, while there were no significant differences in the mean normal liver volume and that irradiated with < 20 gray relative biological effectiveness (GyRBE).

Forty-seven patients (28.1%) experienced significant elevations in bilirubin levels after PBT completion (Table S1). The median interval between PBT completion and the onset of significant elevation in bilirubin levels was 7 months (range, 1–16 months). After the event, the normalizations of bilirubin level were observed in 20 patients among those patients. Among 47 patients, 27 and 10 experienced significant elevations in bilirubin levels due to intrahepatic disease progression and subsequent treatments, respectively. Finally, a PBT-related significant elevation in bilirubin levels occurred in seven patients (4.2%), all of whom had underlying liver cirrhosis (LC). Hepatic failure after LC decompensation following PBT was observed in five patients, three of whom died of hepatic failure; two were lost to follow-up after supportive care. The remaining two patients underwent liver transplantation; of these, one patient finally died of subsequent extrahepatic progression and one was lost to follow-up after supportive care. Multivariable analysis revealed that ALBI grade 2 or 3 before PBT was a significant risk factor for PBT-related biliary complications, while PBT dose and tumor location were not (Table 2). The crude rates of PBT-related complications in the ALBI grade 1 and 2, 3 groups were 2.2% and 14.3%, respectively (*p* = 0.016).

The details of newly appeared radiologic biliary abnormalities after PBT completion are summarized in Table 3. Radiologic biliary abnormalities developed in 12 patients (7.2%): 12.5% of patients in the perihilar group and 2.3% of patients in the non-perihilar group; this difference was not statistically significant (*p* = 0.149). Biliary obstructions in the PBT field occurred in seven patients, all of whom experienced causative local tumor progression. The remaining five patients showed biliary obstructions outside the PBT field caused by intrahepatic disease progression (*n* = 2) and other treatments (*n* = 3; Table 3). Therefore, we observed no PBT-related radiologic biliary abnormalities.

**Figure 1:**
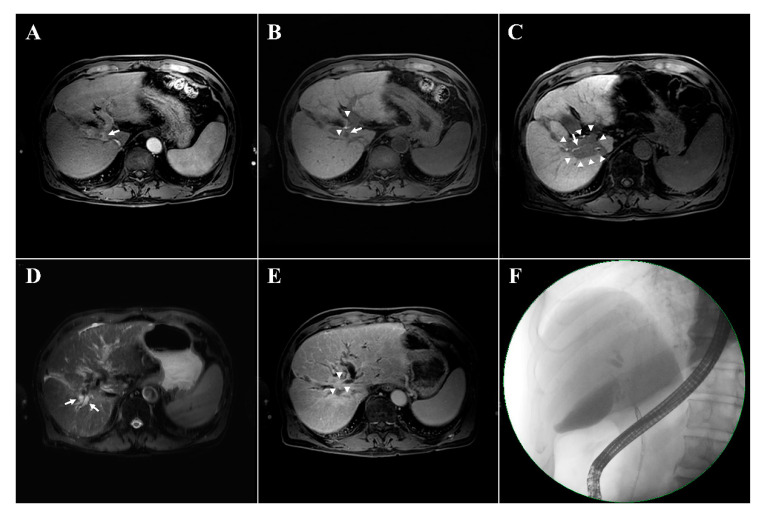
Examples of newly developed radiologic biliary dilatation after the proton beam therapy in a 72-year-old male patient with hepatocellular carcinoma (HCC). The magnetic resonance imaging (MRI) shows the HCC with arterial enhancement in the segment 1 (arrow, **A**). In the hepatobiliary phase, contrast filling is visible in the biliary tract (arrowheads, **B**) and the HCC shows contrast washout (arrow, **B**). A focal liver reaction appears in the irradiated area (arrowheads, **C**) with contrast filling maintained in the hepatobiliary phase (arrow, **C**). Biliary tract dilatation appeared 14 months after the completion of proton beam therapy (arrows, **D**) with obstruction (arrowheads, **E**) and contrast filling defects in the hepatobiliary phase (**E**). No evidence of recurrent disease is seen on MRI (**D**,**E**). Endoscopic retrograde cholangiopancreatography confirming the presence of biliary dilatations with biliary stricture (**F**). The patient underwent liver transplantation due to liver function deterioration; histological examination revealed tumor progression with periductal infiltrating growth that was not detected radiologically.

Figure 1 presents an example of patient who showed a newly developed radiological biliary obstruction underwent subsequent liver transplantation; pathologic examination revealed local tumor progression that had not been observed radiologically (Figure 1). 

**Figure 2:**
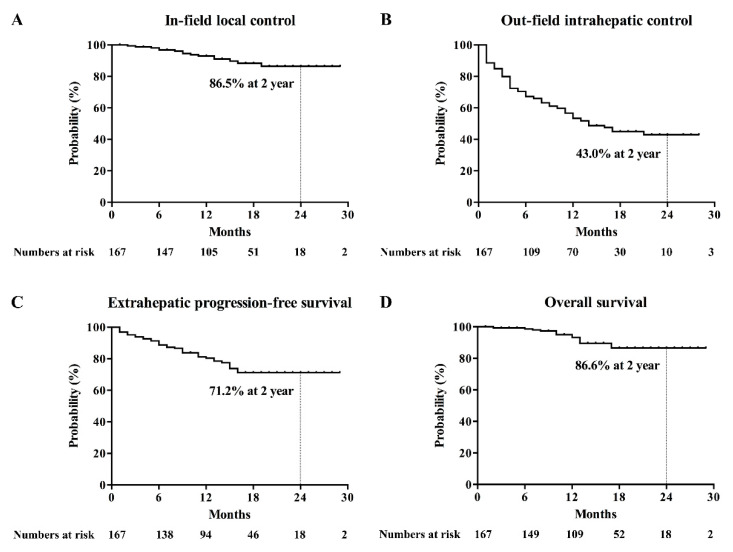
Kaplan–Meier’s curves for (**A**) in-field local control, (**B**) out-field intrahepatic control (**C**), extrahepatic progression-free survival, and (**D**) overall survival.

Other complications were observed, including grade 1 (*n* = 16) and grade 2 (*n* = 3) radiation dermatitis, grade 2 chest wall myositis (*n* = 3; 1.8%), grade 3 gastroduodenal ulcer (*n* = 2, 1.2%), grade 1 pleural effusion (*n* = 14; 8.3%), grade 1 radiation pneumonitis (*n* = 24; 14.4%), classic radiation-induced liver disease (RILD; *n* = 10; 6.0%), and non-classic RILD (*n* = 2; 1.2%).The actuarial rates of in-field local control (IFLC), out-field intrahepatic control (OFIHC), extrahepatic progression-free survival (EHPFS), and overall survival (OS) at 2 years were 86.5%, 43.0%, 71.2%, and 86.6%, respectively (Figure 2). The complete response (CR) rate at 2 years was 87.9% (Figure S1). The multivariable analysis revealed that a maximum tumor diameter of 2 cm or less was an independent prognostic factor associated with high rates of IFLC and EHPFS (Table 4). Early CR was identified as an independent prognosticator for OFIHC and EHPFS (Table 4).

## 3. Discussion

For long-term survival, local tumor control is essential for patients with HCC, even if intrahepatic metastasis is the main failure pattern after the local treatment of primary HCC [16]. Although surgical resection or RFA achieves excellent local tumor control, the eligibility depends on patient condition and tumor characteristics, including size and location [3]. In addition, TACE shows unsatisfactory local tumor control rates [17,18]. RT has contributed to improved local control in the treatment of primary HCC by aiding or as an alternative to standard local modalities, and some guidelines for the treatment of HCC recommend RT as a primary or alternative choice of treatment [5,19,20,21]. As a dose-response relationship has been reported in RT for HCC, there have been efforts to safely deliver high-dose radiation to the primary HCC using advanced RT techniques [6,22,23]. PBT is one of the most advanced forms of RT, and its use in the treatment of primary HCC is expanding [10,22]. The safety of PBT for primary HCC is reliable with regard to RILD or gastroduodenal toxicities after PBT [9]. However, few reports have focused on RT-related biliary complications, especially for PBT [15]. In particular, as active attempts are being made for higher-dose irradiation with PBT, the risk of RT-induced biliary complications, such as biliary stricture and the consequential upsurge of bilirubin levels, may also increase [24].

Early literature reported biliary stricture after RT [12,13]. In those case reports, three of four patients experienced extrahepatic bile duct strictures after abdominal RT without any evidence of recurrent tumor. Histological examinations in two cases showed results consistent with late injuries due to RT performed 10 years before the biliary events [13]. However, those studies involved small-sized case series, and the information on RT was unclear. Although previous animal studies have suggested that radiation-induced bile duct sclerosis and the obliteration of capillary vessels could be the pathogeneses of RT-induced biliary complications [25,26], subsequent studies were seldom conducted. The scarcity of investigations might be due to the low incidence of RT-induced biliary complications [15]. In previous studies, the rates of biliary complications, including biliary stricture, after external beam RT were as low as 1–2%, even with high-dose irradiation for centrally located tumors [14,15]. Moreover, for PBT with relative biological effectiveness higher than that for X-ray RT, PBT-related biliary complications occurred in less than 2% of cases [24].

Contrary to these concerns, perihilar location of the tumor was not a risk factor for PBT-related complications in the present study. Intrahepatic tumor progression, rather than the tumor location, were the main triggers for significant elevation in bilirubin levels or radiologic biliary events. Intrahepatic tumor progression can result in periductal compression or ductal invasion, leading to biliary obstruction. Therefore, tumor progression in the perihilar area is more prone to cause biliary obstruction than non-perihilar progression. In addition, the cause of benign biliary obstruction by PBT itself and tumor progression may be indistinguishable even by careful evaluation of radiological findings, as was the case in Figure 1. Osmundson et al. reported the rates of hepatobiliary toxicity and its association with the dosimetric parameters of central biliary tract volumes after stereotactic body RT to tumors in the central liver [27,28]. The rate of biliary toxicity was above 21%, even though the researchers censored biliary toxicities due to tumor progression. However, in that study, tumor progression was confirmed only based on radiologic findings, which could underestimate the true rate of tumor progression or miss pre-existing intraductal tumors. Therefore, the results should be interpreted carefully, and further investigation is required to improve the distinction between benign biliary obstruction due to RT and true tumor progression.

Rather than the tumor location, ALBI grade, a marker of underlying liver function, was a significant factor associated with PBT-related bilirubin complication in the present study. In addition, all patients who experienced PBT-related bilirubin complications had underlying LC. However, the criteria of PBT-related biliary complication in the present study includes the significant elevation in bilirubin level which also reflects the decline of liver function [8]. The high ALBI grade, which is associated with the poor liver function and a predictor for RILD, could be also identified as a risk factor for the PBT-related biliary complication by increasing the risk of the deterioration of liver function, especially non-classic RILD [29,30]. Therefore, careful selection of dose prescription and beam direction are required to minimize the dose received by the normal liver parenchyma and conserve the liver function when PBT is applied to patients with poor underlying liver function and LC. However, because the progression of perihilar HCC itself potentially increases the risk of biliary events, efforts to improve the local control of perihilar HCC are also necessary to prevent tumor-related biliary obstructions.

Treatment-related biliary complications are an issue not only in RT but also in other local treatments. Biliary complications are generally considered possible sequelae after surgery [31]. RFA can result in direct thermal injury of the bile duct [32]. TACE also frequently causes biliary complications due to ductal cell apoptosis and/or necrosis from hypoxia induced by vessel occlusion [33]. Therefore, the application of these modalities for perihilar HCC has been limited. Considering these limitations of these local modalities, our results imply that PBT is an efficacious and safe local therapy for perihilar HCC. Data from large-scale multi-institutional studies are necessary to generalize our results.

The present study has several limitations. This is a retrospective study with a small sample size from a single institute; therefore, selection bias is possible. Because the etiologies of biliary complications were evaluated clinically and radiologically without histological confirmation, bias was also possible in the evaluation of etiology. In addition, as mentioned above, the criteria of the biliary complication in the present study is incomplete that in some cases the non-classic RILD and the significant elevation of bilirubin are not distinguishable. Therefore, more obvious criteria for clinical biliary complication by PBT is required to be developed. Finally, a longer follow-up duration is necessary to evaluate very long-term complications because, as indicated in early reports, biliary complications can occur even 10 years after treatment [13].

## 4. Materials and Methods

### 4.1. Patients and Treatments

The multidisciplinary team for liver cancer identified candidates for PBT who were not eligible for standard local therapy such as resection or RFA. All patients who were considered candidates for PBT underwent respiratory training and tests of technical eligibility for PBT with voluntary respiratory motion control training a week before the simulation for the further patient selection. Based on the results of the training and testing, the methodologies for respiratory motion control were chosen between breath-hold or regular breathing techniques. The breath-hold technique was preferred as it provides smaller positional uncertainty and temporal changes of locational tissue densities due to organ motion. However, if the breath-hold technique was not suitable, the regular breathing technique was attempted. The regular breathing technique included respiratory-gating PBT covering a limited range of respirations and non-gating PBT encompassing the whole amplitude of respirations. The use of a pencil-beam scanning (PBS) module was considered under the following conditions: (1) the need for dose conformality at the proximal edge where PBS was required rather than passive scattering and (2) large tumor size for which PBS had benefits due to dosimetry homogeneity. The detailed processes for the preparation of PBT were as described previously [10,11]. We retrospectively reviewed 177 patients who were treated with PBT to the primary HCC from January 2016 to December 2017. Among them, we selected 167 patients who were prescribed with 75 GyRBE of biological effective dose with 𝛼/β = 10 (GyRBE_10_) or more. Ten patients were excluded due to uncontrolled lung cancer (*n* = 1), PBT only to portal vein tumor thrombosis without coverage of the whole primary HCC (*n* = 1), loss to follow-up (*n* = 2), or prescribed dose less than 75 GyRBE_10_ (*n* = 6).

### 4.2. PBT Planning Protocol

The protocol of PBT planning for primary HCC was described in the previous literature [10,11]. For the breath-hold technique, the gross tumor volumes (GTV) were delineated on the six sets of computed tomography (CT) images obtained with repetitive breath hold at the same level and summed to cover the deviation of each breath hold. For the regular breathing technique, GTVs were delineated in the non-enhanced 4-dimensional CT images relevant to the amplitude of the gating window or those encompassing the amplitudes of all respiratory phases. By extending as much as 0.5 cm from the union of all delineated GTVs, CTV was generated. The planning target volume was defined with an additional margin of 0.5 cm to the CTV. As the PBT planning image, the second non-enhanced CT image and the maximum intensity projection image generated from the CT scans used for GTV delineation were used in the breath-hold and regular breathing techniques, respectively. The directions, energies, or the aperture shapes of beams were determined to minimize the doses delivered to a normal liver, stomach, bowels, and skin. We used the treatment planning system RayStation (RaySearch Laboratories, Stockholm, Sweden). Basically, PBT dose was determined by the locations of tumor. For the peripheral HCCs far from porta hepatis more than 1 cm, 66 GyRBE in 10 fractions was prescribed, while for those adjacent to the portal hepatis less than 1 cm, 72.6 GyRBE in 22 fractions was prescribed. The dose prescription could be modified according to the purpose of PBT or tolerable dose of organs at risk. The dose constraints of organs at risk are summarized in Table S2.

### 4.3. Complications

The RILD was categorized as classic or non-classic. Classic RILD included anicteric hepatomegaly, ascites, or elevated alkaline phosphatase levels that were more than twice the upper limit of the normal range. Non-classic RILD was defined as the elevation of liver transaminase levels to more than five times the upper limit of the normal range or a worsening of Child-Pugh score by ≥2 points. The criteria for defining PBT-related biliary complications were as follows: (1) a significant elevation in bilirubin level after PBT completion, and/or (2) newly developed radiological biliary abnormalities after PBT completion, and (3) events not caused by HCC progression, comorbidity, or other treatments. Significantly elevated bilirubin levels were defined as the increase in the bilirubin level after the PBT above 3.0 mg/dL and also twice of the baseline level according to the criteria in the previous study [15]. Radiologic biliary abnormalities included the new appearance of biloma or biliary dilation, defined as an increase in ductal diameter to more than twice the previously measured diameter or the new appearance of a biliary tree. The PBT-related biliary complications were evaluated according to whether the treated HCC was in the perihilar location or not. The perihilar location was defined as the 1-cm area extending from the right, left, and common hepatic ducts, including the gallbladder and cystic duct. Other toxicities were scored according to the Common Terminology Criteria for Adverse Events (CTCAE) version 5.0 [34].

### 4.4. Statistical Analysis

The first follow-up evaluation was performed 1 month after PBT completion with imaging studies and laboratory tests then every 2–3 months thereafter. The response after PBT was assessed according to the modified Response Evaluation Criteria in Solid Tumor as recommended in the guidelines from the European Association for the Study of the Liver for the assessment of response after locoregional therapies [35,36]. Additionally, we defined an early CR as that within 4 months of PBT completion. The OS, IFLC, OFIHC, and EHPFS were defined as the intervals from the start of PBT to death, in-field local recurrence, out-field intrahepatic recurrence, and extrahepatic progression, respectively, or the last follow-up if there was no relevant event. Fisher’s exact test and *t*-test were used to compare the distributions of categorical and continuous variables, respectively, between the perihilar and non-perihilar HCC groups. Logistic regression was performed to identify the risk factors associated with significant PBT-related biliary complication. Survival curves were plotted using the Kaplan–Meier method. The survival curves were compared by log-rank tests. Multivariable analysis was performed using Cox regression. Two-sided *p*-values < 0.05 were considered statistically significant. Statistical analyses were performed using IBM SPSS Statistics for Windows, version 22.0 (IBM Corp., Armonk, NY, USA).

### 4.5. Ethical Statement

The institutional review board of Samsung Medical Center approved the present study (protocol number, 2019-08-081) and waived the requirement for informed consent. We performed the present study in accordance with the principles of the Declaration of Helsinki.

## 5. Conclusions

The PBT at the current dose scheme showed a low rate of PBT-related biliary complications regardless of perihilar region involvement, with good oncological outcomes. PBT for the treatment of HCC patients with high ALBI grade and underlying liver cirrhosis requires increased attention to reduce PBT-related biliary complications.

## Figures and Tables

**Table 1:** Baseline characteristics of study patients according to the tumor location.

Variable	Tumor Location		*p*-Value
	Non-Perihilar (%) (*n* = 87)	Perihilar (%) (*n* = 80)	
Age (year)			0.740
<65	29 (33.3)	24 (30.0)	
≥65	58 (66.7)	56 (70.0)	
Gender			0.842
Male	72 (82.8)	65 (81.3)	
Female	15 (17.2)	15 (18.8)	
Underlying liver cirrhosis			1.000
Yes	22 (25.3)	21 (26.3)	
No	65 (74.7)	59 (73.8)	
Child-Pugh class			0.190
A	79 (90.8)	70 (87.5)	
B	8 (9.2)	7 (8.8)	
C	0 (0.0)	3 (3.8)	
ALBI grade			0.023
1	78 (89.7)	61 (76.3)	
2,3	9 (10.3)	19 (23.8)	
ECOG-PS			0.013
0	56 (64.4)	36 (45.0)	
1,2	31 (35.6)	44 (55.0)	
Etiology			0.322
Hepatitis B	67 (77.0)	64 (80.0)	
Hepatitis C	6 (6.9)	9 (11.3)	
Alcoholic	7 (8.0)	2 (2.5)	
Others	7 (8.0)	5 (6.3)	
AFP before PBT			0.121
<200 ng/mL	74 (85.1)	60 (75.0)	
≥200 ng/mL	13 (14.9)	20 (25.0)	
∅_max_ (cm)			0.005
≤2	48 (55.2)	26 (32.5)	
>2	39 (44.8)	54 (67.5)	
Macrovascular invasion			0.001
Yes	16 (18.4)	34 (42.5)	
No	71 (81.6)	46 (57.5)	
BCLC stage			<0.001
0,A,B	70 (80.5)	40 (50.0)	
C,D	17 (19.5)	40 (50.5)	
Beam module			0.062
PS	73 (83.9)	57 (71.3)	
PBS	14 (16.1)	23 (28.8)	
Respiratory control			0.190
Breath-hold	66 (75.9)	51 (63.8)	
Regular breathing with gating	6 (6.9)	6 (7.5)	
Regular breathing without gating	15(17.2)	23 (28.8)	
BED_10_ (GyRBE)			0.017
<105	16 (18.4)	30 (37.5)	
105–110	66 (75.9)	48 (60.0)	
>110	5 (5.7)	2 (2.5)	
Mean clinical target volume (± SD; cc)	97.3 (± 149.6)	345.2 (± 619.9)	<0.001
Mean liver volume (± SD; cc)	1288.7 (± 392.0)	1512.0 (± 679.3)	0.004
Mean normal liver volume * (± SD; cc)	1199.4 (± 328.0)	1197.4 (± 314.1)	0.715
Mean normal liver volume irradiated with < 20 GyRBE (± SD; cc)	1066.7 (± 305.6)	986.3 (± 274.3)	0.285

PS, passive scattering; PBS, pencil beam scanning; ALBI, albumin-bilirubin; ECOG-PS, Eastern Cooperative Oncology Group performance status; AFP, alpha-fetoprotein; PBT, proton beam therapy; Med., median; ∅_max_, maximum diameter; BCLC, Barcelona Clinic Liver Cancer; BED_10_, biological effective dose with *α/β* of 10; GyRBE. gray relative biological effectiveness; SD, standard deviation. * The normal liver volume was defined as that with exclusion of clinical target volume from liver volume.

**Table 2:** Multivariable analysis for PBT-related biliary complication.

Variables	Odd Ratio (Range)	*p*-Value
ALBI grade		0.007
1	1	
2,3	10.453 (1.926–56.722)	
Maximum diameter of HCC (cm)		0.841
≤2	1	
>2	1.205 (0.195–7.445)	
Macrovascular tumor invasion		0.247
Yes	3.977 (0.383–41.246)	
No	1	
Tumor location		0.505
Non-perihilar	1.810 (0.317–10.335)	
Perihilar	1	
Beam module		0.972
Passive scattering	1	
Pencil-beam scanning	1.035 (0.146–7.318)	
BED_10_ (GyRBE)		0.801
<105	1.274 (0.193–8.435)	
≥105	1	

PBT, proton beam therapy; ALBI, albumin-bilirubin; HCC, hepatocellular carcinoma; BED_10_, biological effective dose with *α/β* of 10; GyRBE. gray relative biological effectiveness. Underlying liver cirrhosis was not included in the multivariable logistic regression because the odd ratio diverged to infinity due to the absence of event in the patients without underlying liver cirrhosis.

**Table 3:** Radiological biliary abnormality after the completion of proton beam therapy.

Pt.	Location of Primary HCC	Biliary Change	Location of Obstruction	Interval (Months) *	Relation with IHP	Relation with Other Treatment	Bilirubin Elevation	Further Intervention
1	Non-P	D	in-field	15	Yes	Resection	Yes	No
2	Non-P	D	Out-field	4	No	LT	No	ERBD
3	P	D	In-field	15	Yes	No	Yes	No
4	P	B, D	In-field	7	Yes	No	No	No
5	P	D	In-field	2	Yes	No	Yes	PTBD
6	P	B	Outfield	9	Yes	No	Yes	ERBD
7	P	D	In-field	11	Yes	No	Yes	ERBD
8	P	D	In-field	5	Yes	No	Yes	ERBD
9	P	D	Out-field	8	No	LT	Yes	ERBD
10	P	D	Out-field	9	Yes	No	Yes	PTBD
11	P	D	Out-field	3	No	TACE	Yes	ERBD
12	P	D	In-field	7	Yes	No	Yes	No

Pt., patients; HCC, hepatocellular carcinoma; IHP, intrahepatic progression of disease; P, perihilar; D, dilatation; B, biloma; LT, liver transplantation; ERBD, endoscopic retrograde biliary drainage; PTBD, percutaneous trans-hepatic biliary drainage; TACE, trans-arterial chemoembolization. * Time interval from the completion of proton beam therapy to biliary change development.

**Table 4:** Multivariable analysis for survival curves.

Variable	OS		IFLC		OFIHC		EHPFS	
	HR (95%, CI)	*p* Value	HR (95%, CI)	*p* Value	HR (95%, CI)	*p* Value	HR (95%, CI)	*p* Value
Age (year)		0.152		0.716		0.758		0.110
<65	1		1.228 (0.406–3.712)		1		1	
≥65	2.699 (0.695–10.491)		1		1.078 (0.668–1.741)		1.835 (0.872–3.859)	
Gender		0.462		0.801		0.610		0.353
Male	1		1		1		1	
Female	1.773 (0.386–8.147)		1.235 (0.238–6.405)		1.191 (0.608–2.332)		1.638 (0.578–4.641)	
ALBI grade		0.124		0.825		0.387		0.136
1	1		1		1		1	
2,3	2.627 (0.767–8.999)		1.198 (0.240–5.972)		1.300 (0.717–2.357)		1.844 (0.825–4.120)	
ECOG-PS		0.316		0.312		0.676		0.420
0	1		1		1		1	
1,2	1.784 (0.576–5.526)		1.821 (0.569–5.827)		1.105 (0.692–1.765)		1.327 (0.667–2.640)	
AFP before PBT		0.501		0.614		0.061		0.207
<200 ng/mL	1		1		1		1	
≥200 ng/mL	1.570 (0.422–5.841)		1.440 (0.348–5.958)		1.731 (0.976–3.071)		1.668 (0.753–3.697)	
∅_max_ (cm)		0.908		0.047		0.582		0.004
≤2	1		1		1		1	
>2	1.076 (0.309–3.752)		4.859 (1.020–23.141)		1.149 (0.701–1.885)		3.377 (1.467–7.774)	
Macrovascular invasion		0.103		0.510		0.884		0.749
Yes	3.351 (0.782–14.356)		1.505 (0.446–5.071)		1.045 (0.581–1.879)		1.143 (0.504–2.588)	
No	1		1		1		1	
Tumor location		0.379		0.343		0.803		0.481
Non-perihilar	1		1		1		1	
Perihilar	1.674 (0.531–5.280)		1.737 (0.555–5.411)		1.064 (0.655–1.728)		1.294 (0.632–2.653)	
BED_10_		0.422		0.873		0.145		0.237
<105 GyRBE	1		1.094 (0.363–3.295)		1		1.597 (0.735–3.468)	
≥105 GyRBE	1.679 (0.474–5.956)		1		1.474 (0.874–2.485)		1	
Early CR *		0.290		0.221		0.006		0.036
Yes	1		1		1		1	
No	1.855 (0.591–5.824)		1.987 (0.662–5.963)		1.923 (1.208–3.063)		2.136 (1.053–4.335)	

OS, overall survival; IFLC, in-field local control; OFIHC, out-field intrahepatic control; EHPFS, extrahepatic progression-free survival; HR, hazard ratio; CI, confidential interval; ALBI, albumin-bilirubin; ECOG-PS, Eastern Cooperative Oncology Group Performance Status; AFP, alpha-fetoprotein; PBT, proton beam therapy; ∅_max_, maximum diameter; BED_10_, biological effective dose with *α/β* of 10; GyRBE, gray relative biological effectiveness; CR, complete response. * Early CR is defined as CR within four months after the completion of PBT.

## Supplementary Materials

The following are available online at https://www.mdpi.com/2072-6694/12/9/2395/s1, Figure S1: Actuarial rate of complete response, Table S1: Characteristics of the patients who experienced significant bilirubin elevation after the completion of PBT, Table S2: Doses constraints of organs at risk.
