# Supplementary material for: Do Biliary Complications after Proton Beam Therapy for Perihilar Hepatocellular Carcinoma Matter?
Source: Cancers (Basel). 2020 Aug 24;12(9):2395. doi: 10.3390/cancers12092395 (PMC7565009; doi:10.3390/cancers12092395)
Supplement: Supplementary file 1 [file cancers-12-02395-s001.pdf]

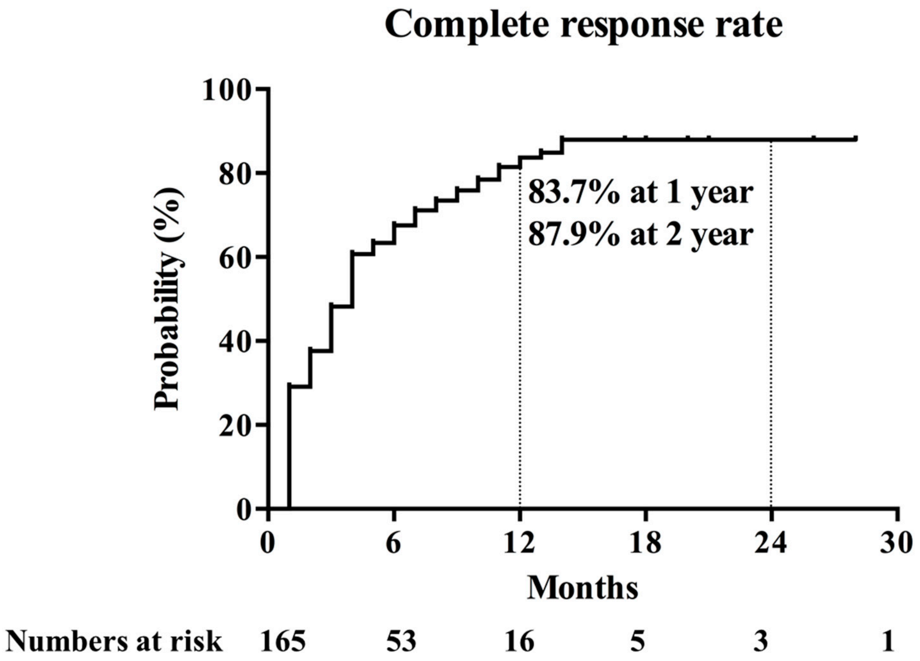

Figure S1. Actuarial rate of complete response

Table S1. Characteristics of the patients who experienced significant bilirubin elevation after the completion of PBT.

#	Location	Underlying LC	Dose (GyRBE) / fx n.	Interval (Month)*	Estimated Causes	Bilirubin Normalization
1	non-perihilar	yes	66 / 10	5	subsequent resection	Yes
2	non-perihilar	yes	50 /10	7	subsequent resection	Yes
3	non-perihilar	yes	50 / 10	6	subsequent resection	Yes
4	non-perihilar	yes	66 / 10	3	subsequent LT	Yes
5	non-perihilar	yes	50 /10	4	IHP	No
6	non-perihilar	yes	66 / 10	12	LC decompensation after PBT	No
7	non-perihilar	yes	66 / 10	12	subsequent RFA	Yes
8	non-perihilar	no	66 / 10	16	IHP	No
9	non-perihilar	yes	66 / 10	13	IHP	Yes
10	non-perihilar	yes	66 / 10	10	Anti-tuberculosis agents	Yes
11	non-perihilar	yes	66 / 10	1	LC decompensation after PBT	No
12	non-perihilar	yes	66 / 10	16	IHP	Yes
13	non-perihilar	no	50 / 10	4	PBT	Yes
14	non-perihilar	yes	66 / 10	5	IHP	No
15	non-perihilar	yes	66 / 10	11	PBT	Yes
16	non-perihilar	yes	66 / 10	9	IHP	No
17	perihilar	no	66 / 10	6	IHP	No
18	perihilar	yes	60 / 10	3	LC decompensation after PBT	No

19	perihilar	yes	66 / 10	13	IHP	Yes
20	perihilar	yes	50 / 10	7	IHP	Yes
21	perihilar	yes	66 / 10	0	IHP	No
22	perihilar	yes	66 / 10	15	IHP	No
23	perihilar	yes	50 / 10	1	IHP	Yes
24	perihilar	yes	50 / 10	5	IHP	No
25	perihilar	yes	50 / 10	2	IHP	Yes
26	perihilar	yes	50 / 10	9	IHP	No
27	perihilar	yes	66 / 10	7	hemolytic anemia	Yes
28	perihilar	no	66 / 10	9	IHP	No
29	perihilar	yes	66 / 10	12	subsequent TACE	No
30	perihilar	yes	50 / 10	5	IHP	No
31	perihilar	yes	50 / 10	15	IHP	Yes
32	perihilar	yes	66 / 10	4	IHP	No
33	perihilar	yes	66 / 10	16	LC decompensation after PBT	No
34	perihilar	yes	66 / 10	10	IHP	No
35	perihilar	yes	48 / 6	4	IHP	No
36	perihilar	yes	66 / 10	11	IHP	No
37	perihilar	yes	66 / 10	3	IHP	No
38	perihilar	no	72.6 / 22	8	IHP	No
39	perihilar	yes	66 / 10	4	LC decompensation after PBT	No

40	perihilar	no	66 / 10	2	subsequent TACE	Yes
41	perihilar	yes	66 / 10	2	hepatitis-B viral flare	No
42	perihilar	yes	66 / 10	8	subsequent LT	Yes
43	perihilar	yes	60 / 5	8	subsequent liver LT	Yes
44	perihilar	yes	66 / 10	8	subsequent LT	Yes
45	perihilar	yes	60 / 5	2	IHP	No
46	perihilar	yes	66 / 10	7	IHP	No
47	perihilar	yes	66 / 10	7	IHP	No

LC, liver cirrhosis; GyRBE, Gray relative biological effectiveness; LT, liver transplantation; IHP, intrahepatic progression of disease; PBT, proton beam therapy; RFA, radiofrequency ablation; TACE, trans-arterial chemoembolization. * This was calculated as the time interval from the completion of proton beam therapy to the onset of significant bilirubin elevation.

Table S2. Doses constraints of organs at risk.

Organs at Risk	Allowed Dose
Spinal cord	$D_{max} \leq 39$ GyRBE
Gastroduodenum	$V_{35\ Gy} < 2cc$
Large bowel	$D_{max} < 40$ GyRBE
Normal liver*	$V_{\geq 20\ GyRBE} < 700$ cc
Skin	$D_{max} < 40$ GyRBE

GyRBE, Gray relative biological effectiveness.

* The normal liver volume was defined as that with exclusion of clinical target volume from liver volume.
